# Funisitis Predicts Poor Respiratory Outcomes in Extremely Preterm Neonates

**DOI:** 10.3390/children12111506

**Published:** 2025-11-06

**Authors:** Yi-Li Hung, Chung-Min Shen, Wu-Shiun Hsieh

**Affiliations:** 1 Department of Pediatrics, Cathay General Hospital, Taipei 106, Taiwan; cgh11534@cgh.org.tw (Y.-L.H.); cgh09218@cgh.org.tw (C.-M.S.); 2 School of Medicine, National Tsing Hua University, Hsinchu 300, Taiwan; 3 Department of Pediatrics, National Taiwan University Children’s Hospital, National Taiwan University College of Medicine, Taipei 100, Taiwan; 4 School of Medicine, Fu Jen Catholic University, New Taipei City 242, Taiwan; 5 Department of Pediatrics, College of Medicine, Kaohsiung Medical University, Kaohsiung 807, Taiwan

**Keywords:** funisitis, chronic lung disease, histological chorioamnionitis, preterm neonates

## Abstract

**Highlights:**

**What are the main findings?**

• Neonates with histological chorioamnionitis (HCAM) and funisitis had a lower gestational age and a higher incidence of clinical chorioamnionitis.
• Extremely preterm neonates with funisitis were associated with a markedly greater protection effect on respiratory distress syndrome but a higher risk to develop early cystic-interstitial lung and chronic lung disease than HCAM.

**What are the implications of the main findings?**

• Extremely preterm neonates with funisitis will have poor respiratory outcomes. The identification of predictive approaches for funisitis and the timely initiation of anti-inflammatory therapy may contribute to improved respiratory outcomes

**Abstract:**

**Background/Objectives:** Histological chorioamnionitis (HCAM) is a risk factor of chronic lung disease (CLD) in preterm neonates. Funisitis, an indicator of fetal inflammatory response, has been linked to adverse neonatal outcomes, but its impact on respiratory outcomes in extremely preterm neonates remains uncertain. In this study, we investigated whether HCAM with funisitis is associated with poorer respiratory outcomes when compared with HCAM alone in preterm (gestational age 22–36 weeks) neonates. **Methods:** This was a retrospective cohort study. We divided very low-birth weight (VLBW) preterm neonates with placenta histopathology examinations into three groups—normal, isolated HCAM, and HCAM with funisitis. Perinatal characteristics, radiographic findings, morbidities, and respiratory outcomes were compared. **Results:** Among 244 VLBW neonates, 25 (10.2%) had HCAM with funisitis, 88 (36.1%) had isolated HCAM, and the remaining 131 were in the normal group. Neonates with HCAM and funisitis had a significantly lower gestational age (26.44 ± 2.1 weeks) but a higher incidence of clinical chorioamnionitis (40.0%) than those with isolated HCAM (12.5%) or normal placentas (6.9%). Moreover, the incidence of cystic–interstitial lung changes before 2 weeks of postnatal age was higher in the HCAM with funisitis group (56.5%) than in the isolated HCAM group (25.0%), and the normal group (4.4%). CLD occurred in 66.7%, 37.7%, and 1.3% of these groups, respectively, and the need for home oxygen at follow-up was 26.1%, 13.7%, and 6.4%. Both isolated HCAM and HCAM with funisitis protected against severe respiratory distress syndrome. However, extremely preterm birth and funisitis had a more adverse impact on CLD development than HCAM alone (adjusted odds ratio 15.259 vs. 3.841). **Conclusions:** Funisitis independently predicts poor respiratory outcomes in extremely preterm infants. The long-term clinical impacts of funisitis in preterm infants should be further investigated.

## 1. Introduction

Chorioamnionitis (CAM) is an ascending infection of the intrauterine cavity during pregnancy [1]. Compared with clinical CAM, histologic chorioamnionitis (HCAM) provides a more reliable histopathologic diagnosis of intra-amniotic infection, with a higher negative and acceptable positive predictive value [2]. The incidence of HCAM reaches 60–80% in extremely preterm neonates, reflecting its high prevalence in this population [3].

HCAM involves inflammation of the placenta, fetal membranes, and umbilical cord, and is classified as isolated HCAM or HCAM with funisitis [4]. Isolated HCAM, characterized by neutrophil infiltration along the amniochorionic plate, represents a maternal inflammatory response to infection and is staged by the depth of neutrophil invasion [4]. In contrast, funisitis denotes inflammation of the umbilical vessels and Wharton’s jelly, representing the fetal inflammatory response syndrome (FIRS) and typically associated with elevated fetal interleukin-6 (IL-6) levels [1,5]. Clinically, the presence of funisitis indicates intrauterine infection extending to the fetal compartment and may predict systemic inflammation and adverse neonatal outcomes.

Exposure to antenatal inflammation has been linked to altered fetal lung development and adverse respiratory outcomes. HCAM and funisitis represent distinct yet overlapping inflammatory pathways—maternal and fetal, respectively—and the presence of funisitis reflects a more advanced stage with systemic involvement. Watterberg et al. first reported that chorioamnionitis was associated with a higher risk of chronic lung disease (CLD) but a lower risk of respiratory distress syndrome (RDS), suggesting that prenatal inflammation may accelerate lung maturation while increasing susceptibility to postnatal injury [6]. However, subsequent studies have reported inconsistent results [7,8], likely due to variations in study design, outcome definitions, and lack of stratification by HCAM and funisitis.

Despite increasing evidence linking intrauterine inflammation with bronchopulmonary dysplasia (BPD), the specific contribution of the fetal inflammatory response, reflected by funisitis, remains unclear, particularly in very-low-birth-weight (VLBW) and extremely preterm neonates [9,10,11].

We hypothesized that funisitis is associated with an increased risk of CLD and other adverse respiratory outcomes, whereas isolated HCAM may exert a distinct, less detrimental effect. Therefore, this study aimed to compare the clinical characteristics and respiratory outcomes—including RDS, early cystic–interstitial lung changes, and CLD—among VLBW preterm neonates with isolated HCAM, HCAM with funisitis, and normal placentas

## 2. Materials and Methods

### 2.1. Study Design and Outcomes

In this retrospective study, we enrolled VLBW preterm neonates (<1500 g birth weight) admitted to the NICU of Cathay General Hospital in Taiwan between June 2007 and August 2020. Neonates with chromosomal abnormalities, major or lethal congenital anomalies, or incomplete placental pathology data were excluded from the analysis. Neonates with chromosomal or major congenital anomalies were excluded. The hospital ethics committee approved the study (CGH-P110067).

Electronic medical records were extracted and reviewed for sex, GA, birth weight, 5 min Apgar score, delivery mode, twin status, prenatal steroid use, and clinical outcomes. GA was determined primarily by first-trimester ultrasound measurement; when unavailable, it was estimated from the last menstrual period and verified by postnatal assessment using the Ballard scoring system. Clinical outcome included early-onset sepsis, hemodynamically significant patent ductus arteriosus (hsPDA) requiring treatment [12], retinopathy of prematurity (ROP ≥ Stage III), necrotizing enterocolitis (NEC, Bell’s stage II–III), intraventricular hemorrhage (IVH ≥ grade III), periventricular leukomalacia (PVL), and mortality. Maternal data included clinical CAM, preterm premature rupture of membranes (PPROM > 18 h), pre-eclampsia, and gestational diabetes mellitus (GDM). Maternal WBC counts and C-reactive protein (CRP) levels before delivery and neonatal WBC counts, absolute neutrophil/lymphocyte counts after birth and CRP levels at 1 day old were collected. Neonatal WBC counts were GA-adjusted using observed-to-expected mean ratios [13].

We focused on respiratory outcomes among neonates with isolated HCAM, HCAM with funisitis, or normal placentas. Primary outcomes included RDS (≥grade III), requiring ventilation and surfactant, and CLD, defined as oxygen dependency at 36 weeks’ postmenstrual age following initial RDS, according to the NICHD consensus definition and early cystic-interstitial lung changes (streaky infiltrates, hyperinflation, and small cysts within 7–10 days, persistent on radiograph) [14]. Secondary outcomes were pneumothorax, persistent pulmonary hypertension of the newborn (PPHN), duration of ventilation/oxygen support, home oxygen use, and CLD or death.

### 2.2. Placenta Histology

Placentas were formalin-fixed and examined under the supervision of three independent pathologists, who were trained in perinatal pathology and followed the diagnostic criteria proposed by Redline et al. and were blinded to the clinical outcomes of enrolled neonates [4]. Five tissue samples (cord insertion site, margins, central placenta, cord, and membranes) were paraffin-embedded, sectioned (1.5 μm), and H&E-stained. Microscopy was employed to assess inflammation in the amnion, chorion, subchorion, decidua, intervillous space, membranes, umbilical cord, and vessels. HCAM was staged by PMN infiltration: Stage 1 (subchorionitis/chorionitis), Stage 2 (chorioamnionitis), Stage 3 (necrotizing CAM). Funisitis was diagnosed by PMN infiltration of umbilical vessel walls or Wharton’s jelly [15]. Internal consensus meetings were regularly held to ensure interobserver consistency.

### 2.3. Statistical Analysis

The enrolled neonates were divided into three groups according to their placental pathology report: normal group (no HCAM or funisitis), isolated HCAM group (HCAM without funisitis), and funisitis group (HCAM and funisitis). Data were expressed as mean ± SD or n (%). The normality of continuous variables was tested using the Shapiro–Wilk test. Normally distributed data were compared using one-way ANOVA with Bonferroni post hoc correction, whereas non-normally distributed data were analyzed using the Kruskal–Wallis test. Categorical variables were compared using chi-square or Fisher’s exact test.

Multivariate logistic regression analyses were performed to assess the independent effects of HCAM and funisitis on respiratory outcomes (RDS and CLD), adjusting for gestational age, prenatal steroid use, twin status, preeclampsia, and PPROM. The results are reported as adjusted odds ratios (aORs) with 95% confidence intervals (CIs). Survival analysis was conducted using the log-rank test. Statistical analyses were performed using SPSS software (version 16.0; SPSS Inc., Chicago, IL, USA). A two-tailed *p* < 0.05 was considered statistically significant.

## 3. Results

During the study period, 319 VLBW neonates (<1500 g) were admitted to the NICU. Three of them with multiple congenital anomalies and 72 (22.6%) with missing placental pathology data were excluded. A total of 244 neonates were analyzed (mean GA: 28 ± 3.2 weeks; mean birth weight: 1040 ± 301 g). Males comprised 55.7% (*n* = 136). Perinatal characteristics and outcomes were comparable between included and excluded neonates.

**Figure 1:**
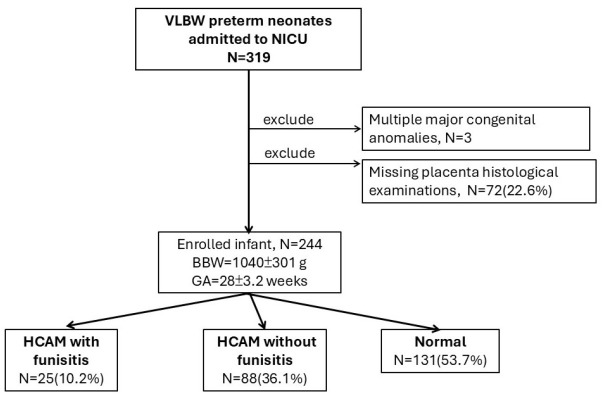
Study participant flowchart.

Of the 244 VLBW preterm neonates, 131 (53.7%) had normal placental pathology results without HCAM or funisitis. Twenty-five neonates (10.2%) had HCAM with funisitis. The remaining 88 (36.1%) neonates had isolated HCAM without funisitis (Figure 1).

The incidence of funisitis was higher in VLBW neonates of smaller GA. Of the extremely preterm neonates less than GA 28 weeks, 17 (14.9%) had funisitis, whereas only 8 (6.2%) neonates with GA ≥ 28 weeks had funisitis. Among neonates with funisitis (n = 25), 3 had early-stage (stage 1) HCAM, 16 had intermediate-stage (stage 2) HCAM, and 6 had advanced-stage (stage 3) HCAM.

Comparing the perinatal characteristics of these three groups [normal: HCAM (+) and funisitis (−); funisitis (+)], we found that neonates with funisitis had a significantly smaller GA and a higher incidence of clinical CAM than neonates with isolated HCAM or normal groups. There were no significant differences in sex, BBW, delivery mode, or antenatal steroid use among the three groups. Neonates with isolated HCAM or funisitis were more likely to have a maternal history of PPROM but exhibited a lower rate of pre-eclampsia (Table 1).

Neonates with funisitis had higher WBC and PMN counts postnatally than those with isolated HCAM or normal placenta (Figures S1A and S2A). After adjusting for GA, the corrected WBC counts remained higher in the funisitis group (Figure S2B). Maternal WBC counts before delivery were higher in the isolated HCAM and funisitis groups than in those without HCAM (Figure S1B). The value of neonatal CRP was different among the three groups but neonates with funisitis did not have higher CRP levels than neonates with isolated HCAM (Figure S3A). However, maternal CRP levels were higher in the isolated HCAM or funisitis groups than in the normal group, with an average CRP level of approximately 2 mg/dL (Figure S3B).

In the unadjusted analysis, neonates with funisitis had significantly higher rates of CLD (66.7%) and early cystic–interstitial lung changes (56.5%) compared with those with isolated HCAM (37.7% and 25.0%) and normal placentas (1.3% and 4.4%) (Table 2). The incidence of RDS did not differ significantly among groups.

**Figure 2:**
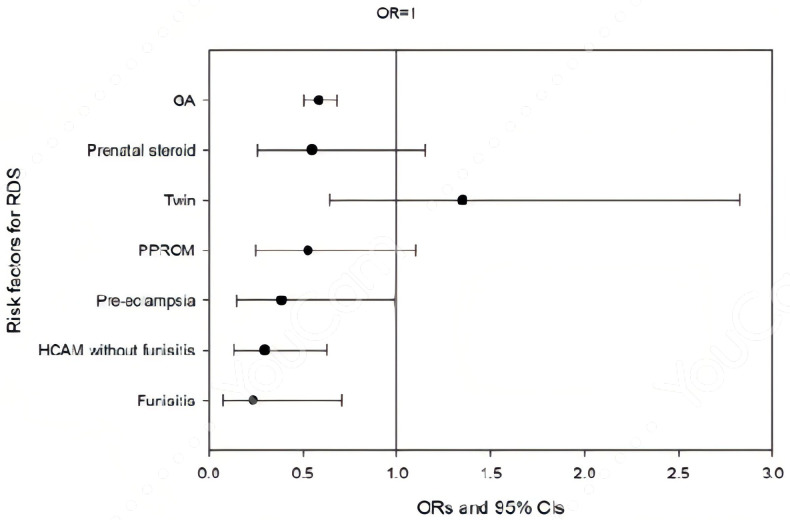
Risk factor multivariate analysis for respiratory distress syndrome (RDS) in very-low-birth-weight (VLBW) preterm neonates. Four factors were significantly and independently associated with RDS: GA (aOR = 0.584; 95% CI 0.504–0.678, *p* < 0.001); maternal history of pre-eclampsia (aOR = 0.384, 95% CI 0.150–0.985, *p* = 0.046); isolated HCAM (aOR = 0.294, 95% CI 0.137–0.631, *p* = 0.002), and funisitis (aOR = 0.233, 95% CI 0.077–0.711, *p* = 0.011). RDS: respiratory distress syndrome; GA: gestational age; PPROM: preterm premature rupture of membrane; HCAM: histological chorioamnionitis; CI: confidence interval; OR: odds ratio.

**Figure 3:**
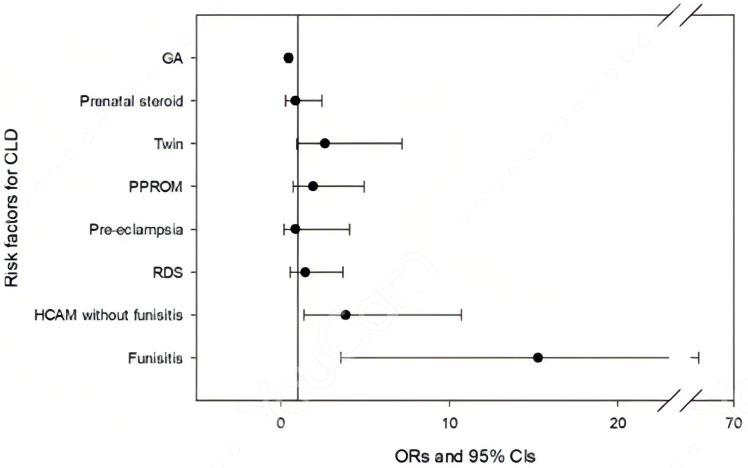
Risk factor multivariate analysis for chronic lung disease (CLD) in very-low-birth-weight (VLBW) preterm neonates. Three factors were significantly and independently associated with CLD: GA (aOR = 0.463, 95% CI 0.356–0.601, *p* < 0.001); isolated HCAM (aOR = 3.841, 95% CI 1.376–10.722, *p* = 0.01), and funisitis (aOR = 15.259, 95% CI 3.573–65.171, *p* < 0.001). CLD: chronic lung disease; GA: gestational age; RDS: respiratory distress syndrome; HCAM: histological chorioamnionitis; PPROM: preterm premature rupture of membrane; OR: odds ratio; CI: confidence interval.

After adjusting for gestational age and other covariates (prenatal steroid use, twin status, pre-eclampsia, and PPROM), multivariate logistic regression revealed that both isolated HCAM and HCAM with funisitis were independent protective factors against severe RDS (aOR = 0.294, 95% CI 0.137–0.631, and aOR = 0.233, 95% CI 0.077–0.711, respectively) (Figure 2). GA and pre-eclampsia were also associated with RDS (aOR = 0.584; 95% CI 0.504–0.678; aOR = 0.384, 95% CI 0.150–0.985). Conversely, neonates with isolated HCAM had an increased risk of CLD (aOR = 3.841, 95%CI 1.376–10.722), and the association was even stronger in those with funisitis (aOR = 15.259, 95% CI 3.573–65.171) (Figure 3). Smaller GA is also a significant risk factor (aOR = 0.463, 95% CI 0.356–0.601). Funisitis remained an independent predictor of CLD after adjusting for GA.

Two of 25 neonates with funisitis (8%) had early-onset sepsis caused by *Candida glabrata* and coagulase-negative *Staphylococcus aureus*. Other morbidities (ROP, hsPDA, NEC, IVH, and PVL) did not differ among groups. Survival rates were also similar (log-rank *p* = 0.730).

## 4. Discussion

This retrospective cohort of VLBW preterm neonates examined respiratory outcomes across isolated HCAM, HCAM with funisitis, and normal placentas. After adjustment for GA and confounders, HCAM with funisitis showed greater protection against RDS but higher risk for CLD than isolated HCAM, a comparison rarely explored in prior studies.

Acute funisitis is an inflammatory reaction of the umbilical vessels and Wharton’s jelly, serving as a histologic marker of the fetal inflammatory response syndrome (FIRS) [2]. Its reported incidence in preterm neonates ranges from 11% to 44% and is more common in spontaneous preterm labor or PPROM than in term births [5,16,17,18,19,20]. Park et al. and Choi et al. further demonstrated that funisitis and fetal inflammatory responses were more severe in spontaneous preterm labor than in PPROM [18,20]. Choi et al. also noted the highest frequency of funisitis in cervical insufficiency, suggesting that different etiologies of preterm birth are associated with varying patterns of HCAM and funisitis, leading to distinct neonatal outcomes [18].

In our cohort, the incidence of funisitis was 10.2%, lower than previously reported [16,17,18,19]. This difference may reflect our inclusion of all VLBW preterm neonates, including those delivered due to maternal preeclampsia or fetal distress, conditions less related to intrauterine infection. We also found that neonates with funisitis had significantly lower GA and a higher rate of maternal clinical CAM (40%) compared with those with isolated HCAM or normal placentas. Consistent with Kim et al. [5], our findings indicate that funisitis signifies a robust fetal inflammatory response, particularly in the setting of clinical chorioamnionitis and extreme prematurity.

Funisitis also affected neonatal hematologic profiles [21,22]. Kim et al. enrolled 197 preterm neonates with and without funisitis and found that those with funisitis had higher leukocyte and neutrophil counts, higher rates of neutrophilia, and lower RBC counts than those without [21]. Romero et at. demonstrated that WBC and neutrophil counts were increased in FIRS [22]. In our study, we further documented that preterm neonates with funisitis had significantly higher corrected WBC and neutrophil counts than neonates in the isolated HCAM and normal groups and that this WBC stimulation effect was more obvious in neonates than in their mothers. Furthermore, we believe that in cases of funisitis, the intra-amniotic infection increases the concentrations of pro-inflammatory cytokines such as IL-6 in the amniotic fluid. This subsequently induces amniotrophic chemotaxis of neutrophils, which concurrently stimulate the fetal bone marrow response [23]. CRP is a highly sensitive acute systemic inflammation marker controlled by IL-6 [24]. Perrone et al. showed that maternal CRP > 2 mg/dL could be a useful, simple predictor of funisitis in preterm neonates with PPROM [25]. Our results showed that neonates with HCAM or funisitis had significantly higher maternal CRP than that in the normal group with an average CRP > 2 mg/dL, as reported previously [25].

Fetal lung maturation and respiratory outcomes after birth are influenced by the intrauterine infection/inflammatory environment. Some animal studies have documented that intrauterine infection accelerates fetal lung maturation by increasing endogenous corticosteroids, resulting in surfactant protein synthesis [26,27]. HCAM has a protective effect against RDS in preterm neonates, but few studies have discussed the relationship between funisitis and development of RDS [17,28,29,30]. However, in a meta-analysis, Liu et al. showed that funisitis is not correlated with RDS, while moderate HCAM had a protective effect on RSD after stratification [31]. In our study, we found that in preterm neonates, funisitis combined with HCAM was associated with a greater reduction in severe RDS compared with isolated HCAM and normal placentas, after adjusting for major confounders such as GA and antenatal steroid exposure (aOR = 0.233 and 0.294, respectively). This finding is consistent with those of Lee et al. [19] and Lahra et al. [17]. However, this association does not imply a direct causal relationship, and the underlying mechanisms require further investigation.

CLD is a complex, multifactorial disorder resulting from interactions between genetic susceptibility and environmental exposures. The degree of lung immaturity remains the strongest risk factor for CLD, but intrauterine inflammation and infection also contribute to fetal lung programming toward CLD [31,32,33,34]. However, previous studies have reported inconsistent effects of CAM and funisitis on CLD development, likely due to differences in study design, inclusion and exclusion criteria, adjustment for confounders, and variations in antenatal corticosteroid exposure. Antenatal corticosteroids may confound the association between funisitis, HCAM and CLD. It can accelerate pulmonary maturation and reduces the incidence of RDS, but it also modulates inflammatory signaling within the fetal lung. By suppressing cytokine activity and dampening the fetal inflammatory response, antenatal steroid could attenuate or mask the apparent association between funisitis and subsequent CLD. In our study, we adjusted for prenatal steroid use in the multivariate regression analysis to minimize this confounding effect. The association between CAM and CLD among preterm infants was reported in three systemic reviews and meta-analyses [10,11,31]. Villamor-Martinez et al. enrolled 158 studies with 244,096 preterm neonates and confirmed that, clinical CAM or HCAM was associated with BPD at day 28 and CLD at a post-menstrual age of 36 weeks, but this association could be modulated by GA and RDS [10]. However, the term “funisitis” was not used in the search strategy of this meta-analysis, so it remains uncertain whether advanced-stage HCAM or funisitis further increases the risk of BPD/CLD [10]. Liu et al. included 16 observational studies and reported a positive correlation between HCAM/funisitis and moderate-to-severe BPD [31]. By contrast, in another meta-analysis including 30 observational studies, Sarno et al. found that preterm neonates with HCAM showed reduced risk of RDS but it did not affect the development of BPD after adjusting GA [11]. Sarno et al. also performed subgroup analysis regarding the association between HCAM, funisitis and BPD and showed that the presence of funisitis did not affect the risk of BPD [11]. In our study, we demonstrated that both HCAM and funisitis had a significant adverse impact on the development of CLD at PMA 36 weeks, whereas neonates with funisitis had a higher risk of developing early cystic-interstitial lung changes within 2 weeks after birth, with most developing CLD thereafter. CLD was classified into six types by Namba et al. according to the presence of RDS, evidence of intrauterine inflammation, and appearance on chest X-ray [35]. Among these, type III CLD was defined as presence of HCAM/funisitis with typical early cystic lung changes, which accounts for 13.5% of total CLD cases [35]. This type III CLD is considered an anachronism of the origin report of Wilson–Mikity syndrome, and the manifestations were similar to those in preterm neonates with funisitis in our study [36]. However, although the association between funisitis and CLD remained statistically significant after adjustment for gestational age and other covariates in our study, the wide 95% confidence interval indicates substantial uncertainty in the effect estimate. This imprecision likely results from the relatively small number of funisitis cases in our cohort. Therefore, while our findings suggest a strong link between fetal inflammatory response and the risk of CLD, the magnitude of this effect should be interpreted with caution. Larger multicenter studies are warranted to validate this association and provide more precise risk estimates.

Two possible pathways are responsive to CLD induced by HCAM/funisitis. First, HCAM will cause the release of pro-inflammatory cytokines and chemokines into the amniotic fluid and then diffuse into the fetal intra-alveolar space and destroy the pulmonary extracellular matrix directly or indirectly [15,37,38]. Second, funisitis activates fetal neutrophils, releasing systemic inflammatory mediators that increase lung vulnerability to oxidative stress, ventilation, or infection [39]. D’alquen et al. reported that, in preterm neonates, HCAM and funisitis would induce the expression and shedding of the adhesion molecules, such as vascular cell adhesion molecule-1, in the umbilical cord [40]. These upregulated adhesion molecules were associated with increased inflammatory mediators, such as IL-1β, IL-6, and soluble E-selectin, in the fetal circulation [40]. Therefore, rather than isolated HCAM, acute funisitis has a more incremental adverse impact on lung development and is a better histologic marker for CLD in preterm infants. In clinical practice, because preterm infants with funisitis are more likely to develop chronic lung disease early, it is essential to send the placenta for pathological examination immediately after birth to determine the presence of funisitis. This allows for timely initiation of anti-inflammatory treatments, such as hydrocortisone, to prevent the development of chronic lung disease [41].

Our study had some limitations, including its retrospective, single-center design and relatively small sample. Although all placental specimens were reviewed under the supervision of three independent perinatal pathologists following standardized diagnostic criteria, interobserver variability was not formally quantified, which may have introduced minor diagnostic variation. Another limitation is the lack of funisitis staging based on Redline’s criteria [4]. Placental reports recorded only the presence or absence of funisitis, and the small number of cases precluded meaningful subclassification or statistical analysis. This limitation may have attenuated potential dose–response relationships between the extent of umbilical inflammation and neonatal pulmonary outcomes. Matsuda et al. demonstrated that necrotizing funisitis was independently associated with CLD [42]. They suggested that necrotizing funisitis, as an advanced stage of the fetal inflammatory response, represents a distinct pathological process rather than a simple progression of acute funisitis. Therefore, future prospective studies incorporating Redline’s standardized staging system for funisitis are warranted to validate and extend our findings. Finally, only non-specific inflammatory markers, such as white blood cell count and C-reactive protein, were available for analysis in our study. These parameters provide limited mechanistic insight into fetal inflammatory response syndrome. Specific cytokines, including IL-6, IL-8, and TNF-α in amniotic fluid, cord blood, or tracheal aspirates, would offer more direct evidence of intrauterine inflammation. Future prospective studies incorporating cytokine profiling are warranted to clarify the pathophysiologic link between FIRS and subsequent CLD development in preterm infants.

## 5. Conclusions

Funisitis is an independent risk factor for adverse respiratory outcomes in extremely preterm neonates. Early identification may enable targeted surveillance and intervention.

## Figures and Tables

**Table 1:** Perinatal characteristics of neonates with/without HCAM and funisitis.

	Normal N = 131	HCAM (+) Funisitis (−) N = 88	HCAM (+) Funisitis (+) N = 25	*p* Value
**Sex (M:F)**	75:56	50:38	11:14	0.459
GA (weeks)	28.56 ± 3.3	27.10 ± 2.9 ^#^	26.44 ± 2.1 ^#@^	<0.001
BBW (gm)	1074.53 ± 305.8	1002.89 ± 297.6	992.84 ± 280.3	0.160
Vaginal delivery *	12 (9.2%)	14 (15.9%)	5 (20.0%)	0.156
Twin gestation *	48 (36.6%)	25 (28.4%)	1 (4.0%)	0.002
Antenatal steroid use	84 (64.1%)	67 (76.1%)	17 (68.0%)	0.169
Maternal history of PPROM	23 (17.6%)	35 (39.8%)	8 (32.0%)	0.001
Maternal history of pre-eclampsia	38 (29.0%)	4 (4.5%)	1 (4.0%)	<0.001
Maternal history of GDM *	13 (9.9%)	11 (12.5%)	1 (4.0%)	0.528
Clinical chorioamnionitis	9 (6.9%)	11 (12.5%)	10 (40.0%)	<0.001
AS < 7 at 5 min	40 (30.5%)	27 (30.7%)	11 (44.0%)	0.396

Continuous variables are presented as mean ± SD. Categorical variables are presented as numbers (percentage). ANOVA with Bonferroni post hoc analysis and χ^2^–tests were performed for continuous and categorical. Variables, respectively. * Fisher’s Exact test. ^#^ *p* < 0.05 compared with normal group. ^@^ *p* < 0.05 compared with HCAM (+) Funisitis (−) group.

**Table 2:** Respiratory outcomes of VLBW neonates with/without HCAM and funisitis: univariate analysis.

	Normal	HCAM (+) Funisitis (−)	HCAM (+) Funisitis (+)	*p* Value	95% CI
RDS *^#^	65 (49.6%)	38 (43.1%)	12 (48%)	0.643	0.875, 2.467
CLD ^#^	14/114 (1.3%)	29/77 (37.7%)	16/24 (66.7%)	<0.001	2.394, 6.334
Pneumothorax *	20 (15.3%)	10 (11.4%)	2 (8.0%)	0.618	0.385, 1.282
PPHN *^#^	26 (19.3%)	15 (17.0%)	6 (24.0%)	0.704	0.639, 1.643
Early cystic interstitial lung change	5/113 (4.4%)	20/80 (25.0%)	13/23 (56.5%)	<0.001	2.926, 9.478
Mechanical ventilation duration (days)	5.66 ± 11.1	7.44 ± 15.6	12.38 ± 21.5	0.236	4.78, 9.42
O_2_ usage durations (days)	34.63 ± 28.1	48.90 ± 30.4	69.96 ± 39.8	<0.001	38.44, 48.81
Home O_2_ usage *	7/109 (6.4%)	10/73 (13.7%)	6/23 (26.1%)	0.019	1.252, 4.123
CLD or death	33 (25.2%)	40 (45.5%)	18 (72.0%)	<0.001	5.197, 25.324

Continuous variables are presented as mean ± SD. Categorical variables are presented as numbers (percentage). s * Fisher’s exact test. ^#^ VLBW: very low birth weight; HCAM: histological chorioamnionitis; RDS: respiratory distress syndrome; CLD: chronic lung disease; PPHN: persistent pulmonary hypertension of newborn; CI: confidence interval.

## Data Availability

The original contributions presented in this study are included in the article/Supplementary Material. Further inquiries can be directed to the corresponding author.

## Supplementary Materials

The following supporting information can be downloaded at: https://www.mdpi.com/article/10.3390/children12111506/s1, Figure S1: Changes in neonatal and maternal WBC counts. (A) Neonates with funisitis had higher mean WBC counts than those from the isolated HCAM or normal group (25,771 ± 14,053 vs. 10,029 ± 6450 vs. 7608 ± 3250/µL *p* < 0.01) (B) Maternal WBC counts before delivery were higher in the funisitis group and in the isolated HCAM group than in the normal group. (16,282 ± 4656 vs. 14,976 ± 5399 vs. 12,450 ± 4460/µL, *p* < 0.001). Figure S2: (A) Neonatal absolute PMN counts were higher in the funisitis group than in isolated HCAM and normal groups. (2800 ± 2081 vs. 5115 ± 4646 vs. 17,165 ± 11,290/µL, *p* < 0.001) (B) Corrected neonatal WBC by GA was also higher in the funisitis group than in the HCAM group than in normal group. (2.608 ± 1.59 vs. 1.463 ± 1.16 vs. 0.957 ± 0.94, *p* < 0.001). Figure S3: Changes of neonatal and maternal CRP level. (A) Neonates with isolated HCAM had a significantly higher CRP level (0.837 ± 1.282 mg/dL) than neonates with normal placenta (0.325 ± 0.63 mg/dL), but levels were not different in neonate with funisitis. (0.747 ± 0.74 mg/dL) (B) Maternal CRP were both significantly higher in cases with HCAM (2.09 ± 2.4 mg/dL) or funisitis (2.12 ± 1.8 mg/dL) than in normal groups (0.96 ± 1.1 mg/dL).

## Abbreviations

The following abbreviations are used in this manuscript:

HCAM	Histological chorioamnionitis
CLD	Chronic lung disease
VLBW	Very-low-birth-weight
RDS	Respiratory distress syndrome
CAM	Chorioamnionitis
PPROM	Preterm premature rupture of membranes
GA	Gestational age
NICU	Neonatal intensive care unit
BBW	Birth body weight
hsPDA	Hemodynamically significant patent ductus arteiosus
ROP	Retinopathy of prematurity
NEC	Necrotizing enterocolitis
IVH	Intraventricular hemorrhage
PVL	Periventricular leukomalacia
GDM	Gestational diabetes mellitus
PPHN	Persistent pulmonary hypertension of newborn
