# Supplementary figures and images for: Funisitis Predicts Poor Respiratory Outcomes in Extremely Preterm Neonates
Source: Children (Basel). 2025 Nov 6;12(11):1506. doi: 10.3390/children12111506 (PMC12651023; doi:10.3390/children12111506)

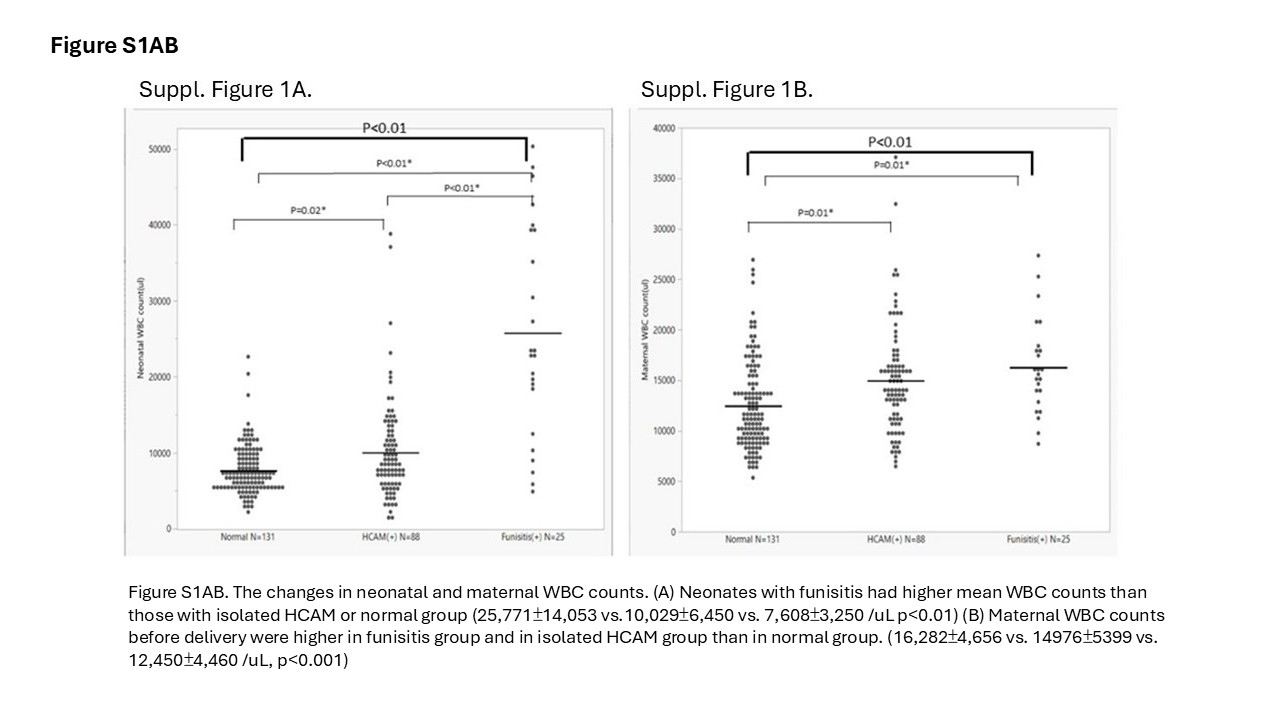

Supplement: Supplementary file 1 [file children-12-01506-s001.zip › Figure S1AB.jpg]

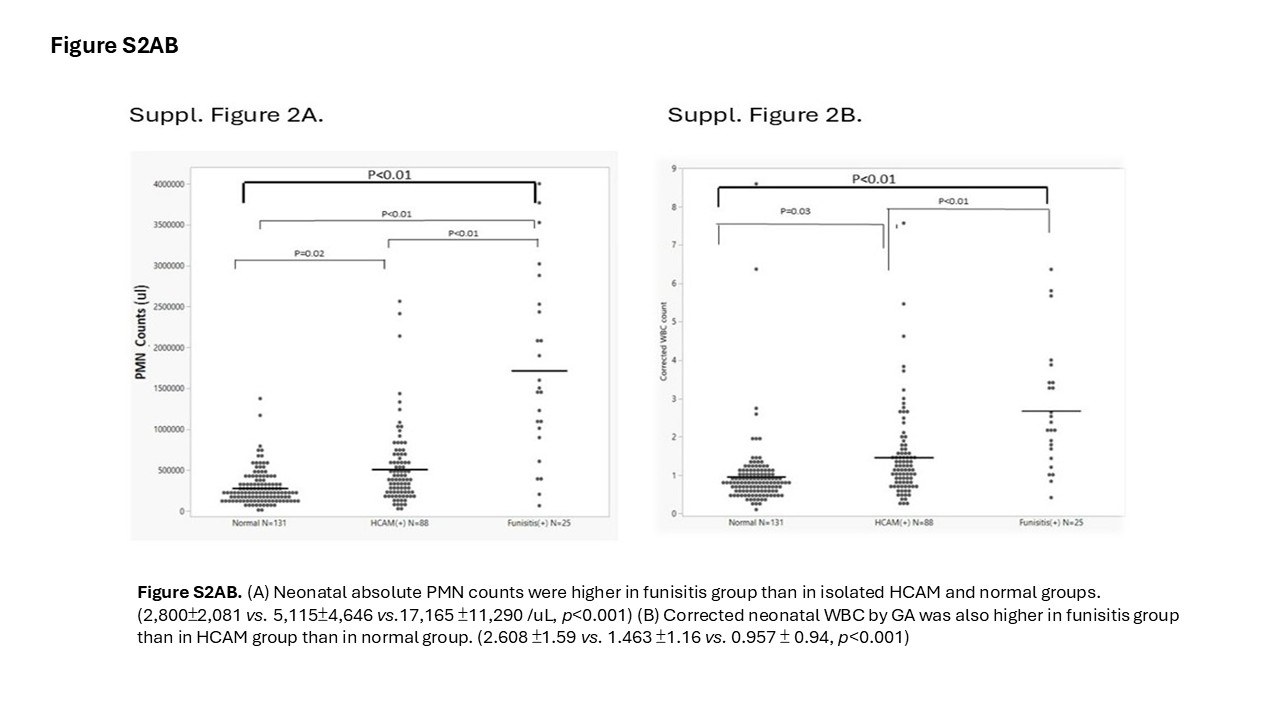

Supplement: Supplementary file 1 [file children-12-01506-s001.zip › Figure S2AB.jpg]

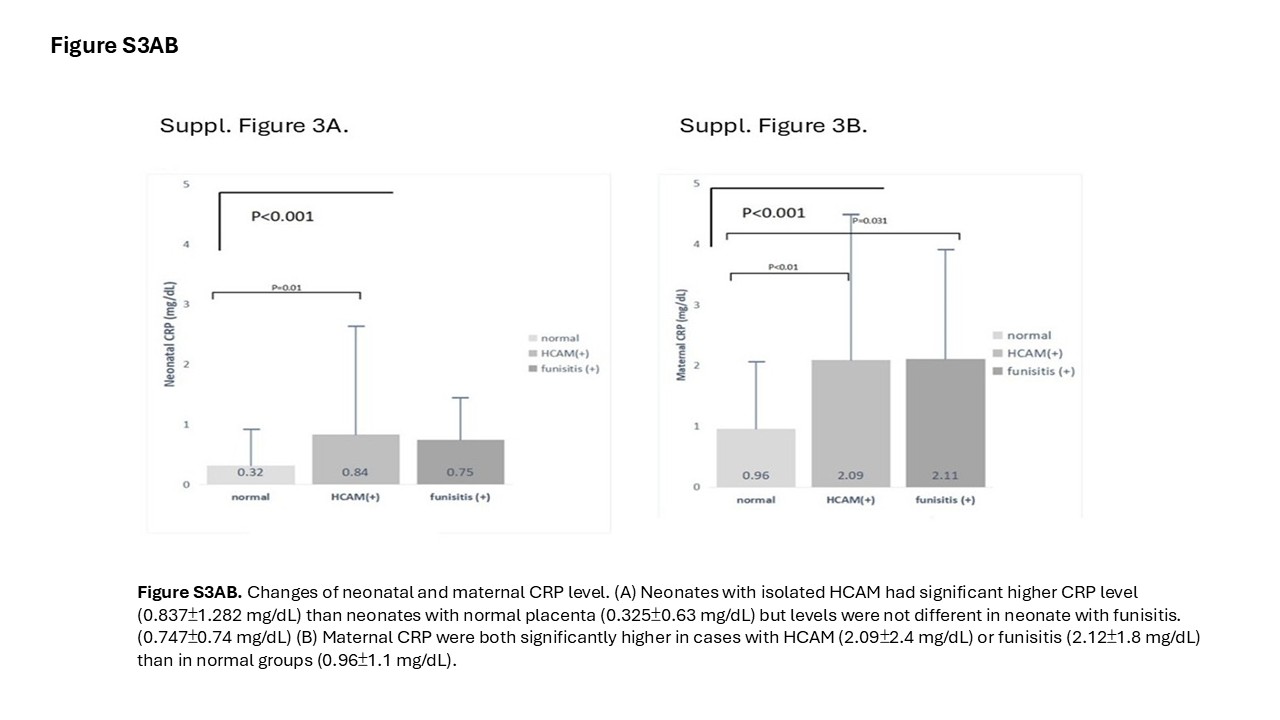

Supplement: Supplementary file 1 [file children-12-01506-s001.zip › Figure S3AB.jpg]
